# Surgical repair of rectus abdominis rupture with rectus sheath hematoma due to chronic cough and prolonged anticoagulant therapy in a COVID-19 patient: A case report

**DOI:** 10.1016/j.ijscr.2024.109628

**Published:** 2024-04-16

**Authors:** Aya Z. Haji Mohamad, Reem M. Kozum, Maria Chakhide, Bashar Tabbakh, Ammar Niazi

**Affiliations:** a Faculty of Medicine, University of Aleppo, Aleppo, Syria; b General Surgery Department, Faculty of Medicine, Aleppo University Hospital, Aleppo, Syria

**Keywords:** COVID-19, Cough, Rectus sheath hematoma, Anticoagulants, Rectus abdominis muscle rupture, Case report

## Abstract

**Introduction and importance:**

Herein, we present an unexpected rectus sheath hematoma (RSH) complication due to chronic COVID-19 related cough and prolonged anticoagulation therapy. COVID-19 usually presents with respiratory symptoms, such as cough. Anticoagulants are used in severe cases of COVID-19 as well as in mechanical heart valve replacement to prevent thrombosis. However, there is a high risk of bleeding.

**Case presentation:**

We report a rare case of a 74-year-old woman who presented with a COVID-19 related cough persistent over two months, and was also undertaking warfarin daily for 10 years due to mechanical mitral valve replacement. Computed Tomography (CT) scan revealed retroperitoneal and rectus sheath hematoma (RSH) as well as rectus abdominis muscle rupture. She had hemorrhagic shock due to rapid hematoma expansion to the right and left flank as well as to the back. Thus, she required an emergency surgery in which the hematoma was excised and the rectus abdominis muscle was sutured. The patient was discharged and has completely recovered.

**Clinical discussion:**

Many factors and mechanisms contribute to the formation of the RSH and the rupture of rectus abdominis muscle, including severe cough and anticoagulants.

**Conclusion:**

Although the use of anticoagulants is necessary for patients who underwent mechanical valve replacement or for COVID-19 patients as a prophylaxis of thrombotic complication, RSH should be kept in mind and carefully monitored as it may require surgical intervention in severe cases.

## Highlights

• Giant left atrium (GLA) is a rare condition often associated with rheumatic heart disease and can lead to cardiac and extra-cardiac complications.
• A 54-year-old woman with a 25-year history of mitral stenosis caused by rheumatic heart disease presented with symptoms of dyspnea, orthopnea, and palpitations.
• The chest X-ray results indicated cardiomegaly and pleural effusion on the right side.

## 1 Background

During the COVID-19 pandemic, cough was the most common symptom accompanied with COVID-19 [1]. It is classified as acute (less than 3 weeks), subacute (3–8 weeks), and chronic (more than 8 weeks); and can result in many complications in different organ systems, such as cardiovascular, gastrointestinal, musculoskeletal, neurologic, and respiratory complications [2].

Thrombosis is one of the most important complications in patients with mechanical heart valves or COVID-19. Therefore, to reduce thrombotic events and mortality rates, many patients with mechanical heart valves undertake lifelong anticoagulation therapy, while COVID-19 patients undertake prophylactic anticoagulants [3,4].

However, both anticoagulants and severe cough predispose patients to high risk of bleeding [3].

We present a rare case of a surgical removal of retroperitoneal and rectus sheath hematoma in COVID-19 patient who presented with chronic cough while on anticoagulation therapy for 10 years. This case was reported according to the SCARE 2023 guidelines [5].

## 2 Case presentation

A 74-year-old woman presented with signs of severe cough and acute abdomen. She had generalized abdominal pain with constipation, and abdominal distention. The physical examination showed peritoneal irritation, and rebound tenderness. She also had bruising and ecchymosis in the right and left flank extended to the back.

Two months prior to admission, she was diagnosed with COVID-19. She had fever, cough with sputum, and was treated with Azithromycin and Dexamethasone. Although she recovered, the cough was gradually increasing throughout the past two months. Two days before the admission, the patient's continuous cough suddenly increased accompanied by a sudden onset of pain in the left flank. The pain transferred to the umbilicus, right flank, epigastrium in order; until it was generalized and accompanied by abdominal distention.

During this period, she lost weight because she could not eat well due to abdominal pain.

The patient does not smoke or drink alcohol. She has 10 children, all normal birth. Her past medical history includes type II diabetes mellitus for two years and atrial fibrillation. She had mechanical mitral valve replacement 10 years ago. As a result, she has been taking warfarin 5 mg since then. The patient Eastern Cooperative Oncology Group (ECOG) score was 3, she had a degree of muscular atrophy due to advanced age.

**Fig. 1:**
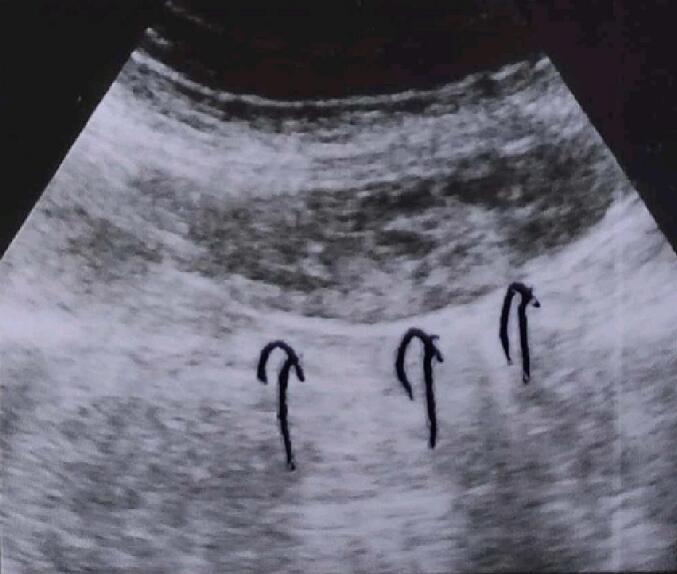
Ultrasound of the abdomen and pelvis, Cystic formation in rectus abdominis.

On admission, the Ultrasound (US) of the abdomen and pelvis showed distended oedematous cystic formation with turbid content in the left and right hypochondria (Fig. 1). Everything else was normal.

**Fig. 2:**
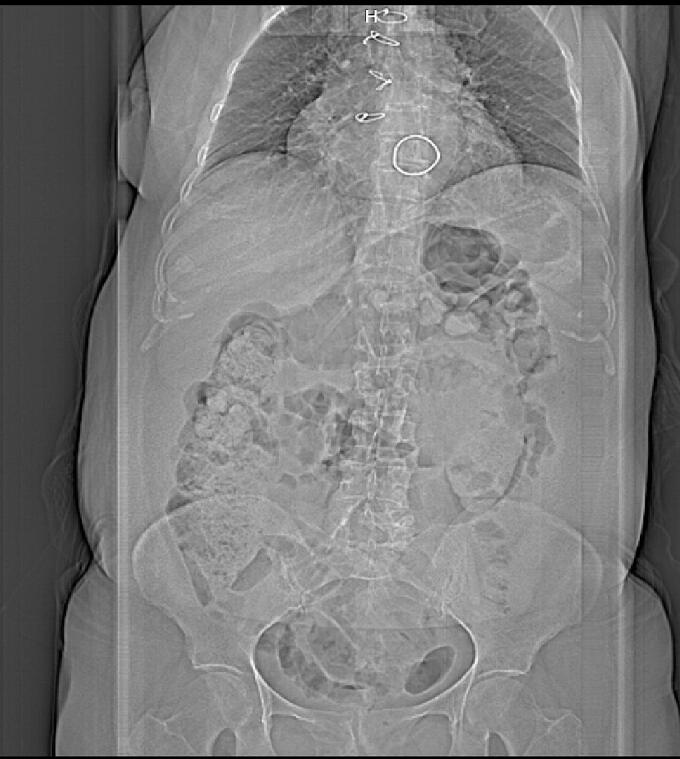
An abdominal X ray, no air was found.

Abdominal X-ray denied gastric or enteric perforation. No free air was found (Fig. 2).

**Fig. 3:**
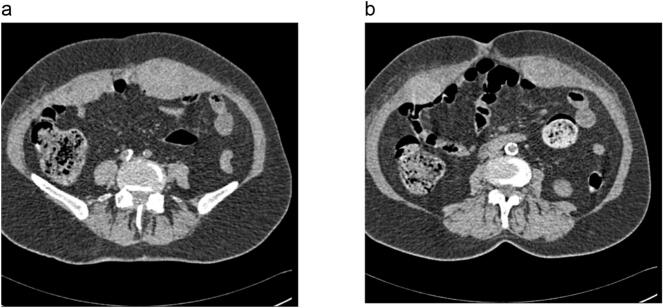
(a, b). CT of the abdomen with a Hematoma of the right and left rectus abdominis muscles.

Computed tomography (CT) scan was performed, and showed asymmetrical increased thickness at both sides of the rectus abdominus and in the posterior wall of the stomach, possibly due to inflammatory infiltration. The maximal thickness was 29 mm and 2–19 mm respectively (Fig. 3). This indicates the presence of retroperitoneal and rectus sheath hematoma (RSH), and rectus abdominis muscle rupture.

Due to the hematoma rapid and continuous expansion, she was hemodynamically unstable and had hemorrhagic shock. She had tachycardia with hypotension. Thus, she needed an emergency surgery. Complete blood count (CBC) showed decreased hemoglobin levels of 8.6 g/dl and hematocrit levels of 25 %; and elevated white blood cells levels of 14,200/cmm, INR of 4.5 and PTTK levels of 40.3 s. As a result, the warfarin was stopped the day before the emergency surgery, and she received 4 units of fresh frozen plasma and one unit of whole blood in order to adjust her INR and hemoglobin levels. In addition, she received paracetamol.

The operation was done by a surgical doctor. A small midline incision was made, and a large hematoma was seen inside the aponeurosis of the left and right rectus abdominis muscles with blood infiltration in the peritoneal cavity. The hematoma was excised, the rupture in the rectus abdominis muscle was sutured, and the abdomen was closed with a drainage tube.

The day after the surgery, she used nasal cannula because her respiratory rate decreased due to abdominal pain. After the surgery, PTTK and INR were prolonged, and she was started on heparin pump 25,000 IU/ml at rate of infusion of 2 ml/h. The heparin pump was gradually stopped the next day and she was back on warfarin. Her general state gradually improved, and she was finally discharged from the hospital.

A week after the surgery, WBC and hemoglobin levels were normal. INR was 2.3.

## 3 Discussion

In light of previous literature; Rectus sheath hematoma (RSH) is a condition that could result either from the rupture of the superior or inferior epigastric arteries or due to the tear of the rectus abdominus muscle. RSH has several risk factors mentioned in the literature including: trauma, surgery, anticoagulation, cough or intense rectus muscle contractions, and pregnancy [6].

As a result of rectus sheath hematoma (RSH), the intraperitoneal bleeding cases are escalating during Covid-19 pandemic. RSH can occur due to the recommended administration of anticoagulation therapy for prophylaxis of thrombotic events that are caused by COVID-19 [7,8]. We identified a summary of these cases in the table below (Table 1). In these cases, most of the patients were female and elderly; most of them presented with different severity of coughing. All patients were on anticoagulation therapy (enoxaparin/warfarin/heparin…) with either prophylactic or therapeutic doses and for different intervals (Table 1). However, none of them presented with rectus abdominis muscle rupture.

**Table 1:** Summary of similar cases.

Case	Age/Sex	Medical history	Clinical presentation	Cough	Diagnosis	Anticoagulation	Intervention	Medical therapy	Outcome
1 [9]	65 years old/Female	Diabetes type 2	Tachycardia and hypogastric pain, huge lower abdominal tender mass.	Non-productive cough (Four days).	Huge rectus sheath hematoma with no extension.	Prophylactic dose of heparin (5000 every 8 h). (For one day)	Angioembolization of the inferior epigastric artery	Isotonic fluids	Alive
2 [7]	78 years old/Female	Diabetes and hypertension.	Revealed a mass in the left lower quadrant.	Acute cough (Three days)	Left inferior RSH approximately 9 cm wide.	Prophylactic dose enoxaparin sodium (40 mg subcutaneously every 12 h) (For nine days).	Non	Fluid replacement, 4 units of erythrocyte suspension (ES), Anticoagulation therapy was stopped.	Alive
3 [10]	54 years old/Female	Mitral valve replacements, stage 4 chronic renal failure, anaemia of chronic disease, diabetes, cholecystectomy, and obesity	Frank, constant sharp pain (intensity: 10/10), marked tenderness with guarding in the hypogastric region and the left upper quadrant of the abdomen.	Episode of severe coughing	10 × 10 × 6 cm hematoma in the left rectus muscle extending into the lower abdomen and extraperitoneal space	Oral anticoagulants (5 mg warfarin daily) (For eight months)	Non	Red blood cells, fresh frozen plasma, and thrombocytes were replaced, ice compression, warfarin was discontinued, low-molecular weight heparin was commenced	Alive
4 [3]	57 years old/Male		Tachypnoeic, no evidence of upper and lower gastrointestinal tract bleeding	Non-productive cough (one week)	Large right retroperitoneal and right psoas muscle hematoma, active bleed within the hematoma.	Prophylactic dose of subcutaneous enoxaparin (40 mg daily) (For 10 days)	Surgical exploration, retroperitoneal hematoma was evacuated, abdominal packing to secure hemostasis, revision surgery (2 days later) abdominal packing was removed	Vasopressor support (48 h), packed cell (14 units), fresh frozen plasma (6 units), cryoprecipitate (12 units), platelets (18 units)	Alive
5 [11]	26 years old/female	No past medical or surgery history	A dense tender mass in right lower abdomen	Dry cough (3 days)	Large right rectus sheath hematoma (about 70 cc), it has increased after 10 days to 90 cc.	Subcutaneous enoxaparin was started 40 mg BID. (5 days)	Non	Enoxaparin was stopped, and without packed blood cell [PBC] infusion.	Died
6 [12]	79 years old/female	Arterial hypertension, diabetes	Abdominal pain, severe anaemia	Not mentioned	Extensive hematoma posterior to the right kidney, another hematoma in the right ileo-psoas muscle of 80 mm diameter	Low-dose heparin. (For 10 days)	Embolization of the intercostal vessels of the 11th and 12th ribs	Several blood transfusions	Not mentioned
7 [13]	63 years old/female	Essential hypertension, rheumatoid arthritis, asthma and previous pulmonary thromboembolism (PTE) (4 months ago).	Pain and feeling of stretching in the left lower quadrant of the abdomen, tenderness on the painful area on palpation	Dry cough for several days, it intensified during treatment.	Rectus sheath hematoma in the left lower quadrant	Therapeutic rivaroxaban (Xarelto 20 mg/day PO) (for 4 months)	Non	Rivaroxaban/Xarelto treatment was discontinued, conservative follow up (reduction in the size was detected)	Alive

In this case; the cause of RSH was due to a combination of several factors. The patient had persistent chronic COVID-19 related cough for 2 months. In addition, she has been taking warfarin daily over a 10-year-interval for the mechanical valve replacement. She also had a degree of muscular atrophy due to advanced age. These factors caused the rupture of the rectus abdominis muscle and the hematoma formation.

Severe episodes of cough resulting from infection have caused very rare cases of diaphragmatic rupture in COVID-19 and COPD patients, although these patients had no anticoagulants intake [14,15]. In addition, anticoagulants intake and cough caused many RSH cases unaccompanied by muscle rupture [16,17] as severe cough could cause a tear in epigastric arteries due to the produced tension [13]. In this case, the patient presented with severe cough for more than 30 days prior to the rupture.

During the cough's forced movement; the abdominal wall muscles contract and cause the diaphragm to move upwards while the ribs are pushed downward, this opposing pressure may cause either dislodgement of the abdominal muscles or a rupture in the muscles similarly to our case. These effects of coughing on the abdomen are rare and require a surgical intervention [18,19].

The most common symptom of RSH is abdominal pain which could be either acute or gradual and is described as sharp, persistent and non-radiating pain; usually associated with a palpable mass, tenderness and abdominal guarding [6,9]. As described, the patient in this case had severe pain that she could barely move or lie down; it started on the left quadrant and shifted to the other side of the abdomen.

RSH has been and is still misdiagnosed in many cases [6,9]. However, serial hematocrit measurements and imaging must be made. Large reductions in hematocrit levels were reported in cases of large RSH,15. US and CT are commonly used to help confirm the diagnosis, with US being the first line diagnostic test in emergency, and CT being more accurate [6,9].

The management of RSH is conservative management as first-line treatment [20]. However, when the patient is hemodynamically unstable then follows embolization (radiology or transcatheter) or surgery in which the hematoma is evacuated; another option is ligation surgery, which involves the ligation of the inferior epigastric artery [21,22]. In this case, the hematoma was rapidly expanding to the right and lefts flank as well as to the back, the patient had hemorrhagic shock with tachycardia and hypotension, thus required an emergency surgery where we excised the hematoma and sutured the rectus abdominis muscle. While in other cases, the bleeding and hematoma were treated with conservative therapy with blood and plasma transfusions and only 2 patients underwent surgical intervention of embolization to control the bleeding of the hematoma (Table 1).

To the best of our knowledge, this is the first reported case of RSH and rectus muscle rupture following severe cough in a COVID-19 patient that was surgically treated in the literature.

## 4 Conclusion

We point out that practitioners should pay extensive attention to each patient with severe cough and on warfarin or other anticoagulants use and consider the rising possibility and risk of the formation of RSH and rupture of the rectus muscle; it is therefore recommended for patients with severe bouts of consistent cough to have limited anticoagulants therapy and be under continuous observation to test prothrombin time (PT) and efficacy of anticoagulants to stay clear of any harmful consequences.

## List of abbreviations

CT: computerized tomography
US: ultra sound
RSH: rectus sheath hematoma
ARDS: acute respiratory distress syndrome
MSOF: multisystem organ failure

## Ethical approval

Not required for case reports at our hospital. Single case reports are exempt from ethical approval in our institution.

## Funding

There is no source of funding for this paper.

## Author contribution

Aya Haji Mohamad: collected the data, wrote the case presentation and reviewed the manuscript.

Reem M. Kozum: wrote the discussion and reviewed the manuscript.

B Bashar Tabbakha: wrote the introduction.

Maria Chakhide: wrote the discussion and is the corresponding author.

Ammar Niazi: reviewed the paper and collected the data.

## Guarantor

Aya Haji Mohamad.

Maria Chakhide.

## Research registration number

Our case report is Not First in Man report.

## Availability of data and materials

All data generated or analysed during this study are included in this published article [and its supplementary information files].

## Patient perspective

The patients participated in the treatment decision and they were satisfied with the results of the treatment. Their perspective on this treatment was to regain the normal functions with good outcomes.

## Consent

Written informed consent was obtained from the patient for publication of this case report and accompanying images. A copy of the written consent is available for review by the Editor-in-Chief of this journal on request.

## Conflict of interest statement

The authors declare that there is no conflict of interest.
